# Clinical Trial: Marine Lipid Suppositories as Laxatives

**DOI:** 10.3390/md10092047

**Published:** 2012-09-20

**Authors:** Orri Thor Ormarsson, Thormodur Geirsson, Einar Stefan Bjornsson, Tomas Jonsson, Pall Moller, Thorsteinn Loftsson, Einar Stefansson

**Affiliations:** 1 Department of Pediatric Surgery, Children’s Hospital, Landspitali-University Hospital, 101 Reykjavik, Iceland; 2 School of Health Science, University of Iceland, 101 Reykjavik, Iceland; Email: einarsb@landspitali.is (E.S.B.); tomasj@landspitali.is (T.J.); pallm@landspitali.is (P.M.); thorstlo@hi.is (T.L.); einarste@landspitali.is (E.S.); 3 Department of Internal Medicine, Landspitali-University Hospital, 101 Reykjavík, Iceland; 4 Department of Surgery, Landspitali-University Hospital, 101 Reykjavík, Iceland

**Keywords:** suppositories, ointment, laxative, omega-3 fatty acid, clinical trial

## Abstract

Cod-liver oil and other marine products containing polyunsaturated fatty acids have anti-inflammatory, anti-bacterial and anti-viral effects and may be useful in the treatment of various inflammatory and infectious diseases. We developed suppositories and ointment with 30% free fatty acid (FFA) extract from omega-3 fish oil. Our purpose was to evaluate the safety of marine lipid suppositories and ointment in healthy volunteers and to explore the laxative effect of the suppositories. Thirty healthy volunteers were randomized either to a study group administrating 30% FFA suppositories and applying 30% FFA ointment to the perianal region twice per day for two weeks, or to a control group using placebo suppositories and ointment in a double blinded manner. Results: No serious toxic effects or irritation were observed. In the study group 93% felt the urge to defecate after administration of the suppositories as compared to 37% in the control group (*P* = 0.001). Subsequently 90% in the study group defecated, compared to 33% in the control group (*P* = 0.001). Conclusion: The marine lipid suppositories and ointment were well tolerated with no significant toxic side effects observed during the study period. The suppositories have a distinct laxative effect and we aim to explore this effect in further clinical trials.

## 1. Introduction

Fish oils are rich in unsaturated fatty acids and approximately 98% of the refined cod-liver oil consists of triglycerides, the rest consisting of unsaponifiable matter, free fatty acids, monoglycerides and diglycerides. The acid part of the glycerides consists mainly of unsaturated fatty acids, including the polyunsaturated omega-3 (*n*-3) fatty acids eicosapentaenoic acid (EPA) and docosahexaenoic acid (DHA) [1]. 

Fish oils such as cod-liver oil containing *n*-3 polyunsaturated fatty acids (PUFA) are thought to have beneficial effects on human health and the interest to use them in pharmaceutical products has grown [2,3,4,5]. An example is the PUFA containing drug Omacor^®^, used to treat hypertriglyceridemia. The effects of polyunsaturated fatty acids on other cardiovascular diseases, inflammatory diseases and various other conditions have been studied in clinical trials [2,5,6,7,8,9,10,11,12,13]. 

Several studies on the anti-viral, anti-bacterial and anti-fungal effects of PUFA have been reported. They show PUFAs to be effective *in vitro* as well as in *in vivo* studies [14,15,16,17,18,19,20,21]. 

Thus PUFA might be useful in the treatment of various infectious and inflammatory diseases in the anal region. We have developed suppositories and ointment made of 30% free fatty acid extract from omega-3 cod-liver oil. The free fatty acid extract was first processed from regular cod-liver oil, but later on from omega-3 fish oil, richer in *n*-3 PUFA. The values of DHA and EPA in the omega-3 fish oil were 18% (% area) and 12% (% area) respectively, as compared to 11% and 8.2% in the regular cod-liver oil. The free fatty acid extract had almost identical fatty acid profile to that of the oil it was extracted from. 

The purpose of this trial was to evaluate the safety of the lipid suppositories and ointment and explore the laxative effect of the suppositories in healthy volunteers.

## 2. Materials and Methods

In the lipid suppositories and ointment we used omega-3 fish oil, free fatty acid extract from omega-3 fish oil and the antioxidant Coviox T70 all provided by Lysi Ltd. (Reykjavík, Iceland). The base for these products consisted of Apifil (bee wax), Precirol ATO 5 (glyceryl palmitostearate), Compritol 888 ATO (glyceryl bihenate), Suppocire NA 0 (Hard fat), all donated by Gattefossé (Saint-Priest, France), and white soft paraffin (vaselinum album). 

The fatty acid composition of the omega-3 fish oil and the free fatty acid extract was almost identical containing a mixture of saturated fatty acids (28%), monounsaturated fatty acids (23%) and polyunsaturated fatty acids (49%) of which 40% were omega-3 fatty acids. The main fatty acids were myristic acid (6.9%), palmitic acid (16.5%), palmitoleic acid (8.5%), stearic acid (3.3%), EPA (19%) and DHA (13%).

The 30% ointment consists of: 300 g omega-3 fish oil, 300 g free fatty acids, 1 g Coviox T70, 49 g Apifil, 20 g Precirol ATO, 330 g vaselinum album. Total 1000 g.

The 30% suppositories consist of: 100 g omega-3 fish oil 300 g free fatty acids, 1 g Coviox T70, 50 g Apifil, 19 g Compritol 888, 530 g Suppocire NA 0. Total 1000 g.

These manufacturing formulas are patent protected. The placebo products were produced with same ingredients excluding fish oil and free fatty acids.

## 3. Clinical Trial

We conducted a double-blinded study with 32 healthy volunteers over 18 years old. After exclusion of non-eligible candidates, *i.e.*, breast feeding, pregnancy, any medications 3 days prior to the study and active ano-rectal disease, 32 healthy volunteers started in the study. Two changed their mind about participating for personal reasons once they had started, and left the study. Thirty finished the study, 15 in each group. 

On day 1 the participants underwent anal examination by an expert physician and were randomized into study group, receiving the active ingredients (suppositories and ointments), and control group, receiving placebo (suppositories and ointments) for a total study period of two weeks. 

The participants administrated the suppositories in the rectum and applied the ointment to the perianal area twice a day with clinical examination by the same expert physician after the first week including anal examination, where any sign of erythema, inflammation, blood or sores were noted. The participants also answered a questionnaire about side effects or irritation on a scale from 1 to 5, where 1 was no irritation and 5 was severe, and if and when they felt any urge for defecation or if and when they defecated (see Table 1). If no toxic reaction was observed they continued with the treatment regimen for the second week, at the end of which they underwent final examination by the same expert physician and answered the same set of questions (see Table 1).

**Table 1:** Clinical symptoms as reported by questionnaire: Itching (scale from 1 to 5 where 1 was non and 5 was severe); Pain (scale from 1 to 5 where 1 was none and 5 was severe); Mucus excretion from anus yes/no; Blood excretion from anus yes/no; Urge for defecation (none, after: 1–5 min, 5–10 min, 10–30 min or longer); Bowel movement (none, after: 1–5 min, 5–10 min, 10–30 min or longer); Disturbing smell (scale from 1 to 5 where 1 was none and 5 was very disturbing).

	Study group week 1 (*n*)	Study group week 2 (*n*)	Control group week 1 (*n*)	Control group week 2 (*n*)
Itching				
1 (none)	14	12	13	13
2	1	3	2	2
3	0	0	0	0
4	0	0	0	0
5 (severe)	0	0	0	0
Pain				
1 (none)	12	12	14	14
2	2	3	1	1
3	1	0	0	0
4	0	0	0	0
5 (severe)	0	0	0	0
Mucus	2	2	1	0
Blood	2	1	0	0
Urge for defecation				
None	1	1	9	10
1–5 min	7	7	1	1
5–10 min	5	7	3	2
10–30 min	1	0	2	2
Longer	1	0	0	0
Bowel movement				
None	1	2	10	10
1–5 min	6	6	0	1
5–10 min	4	4	1	1
10–30 min	2	3	3	3
Longer	2	0	1	0
Disturbing Smell				
1	5	5	14	14
2	5	5	0	1
3	4	2	1	0
4	1	3	0	0
5	0	0	0	0

The subjects randomized to active treatment (3 males and 12 females) had a mean age of 46 years (range 20–71). The placebo group (6 males and 9 females) had a mean age of 43 years (range 18–82). Statistical analysis was performed with SPSS 13.0 for windows. Continuous variables were compared between the groups by Mann Whitney test. All tests were two tailed and *P* < 0.05 was considered significant. Chi square analysis was performed with Fishers exact test to match the data from the questionnaire.

## 4. Results

The anal examinations conducted after week one and two did not reveal any toxic skin reactions in either group. Complaints of itching was not statistically significant different between the groups (13% in both). All complaints were graded minor, or 2 on the scale of 1–5. There were slightly more complaints of mild pain in the study group (20%) compared to the control group (7%) (*P* = 0.129). All complaints were minor and transient. Four complained about noticing a trace of mucus at defecation in the study group as compared to one in the control group (*P* = 0.161). There were three complaints about seeing a trace of blood at defecation in the study group (2 in week 1 and 1 in week 2) as compared to none in the control group (*P* = 0.076). None of the subjects noted both mucus excretion and trace of blood. Other clinical symptoms are demonstrated in Table 1.

**Figure 1:**
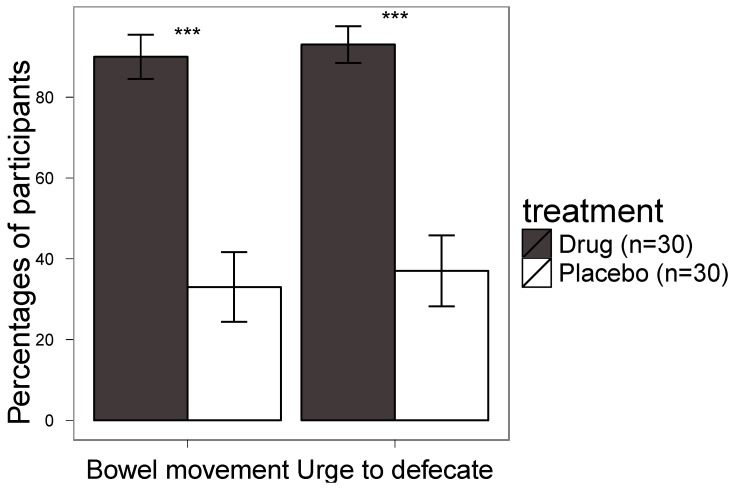
The self reported urge to defecate is shown on the left side and bowel movement after application of suppositories over the 2-week trial period is on the right side. The mean of the one and two week scores is shown. The black columns indicate the study group, which received marine lipid suppositories and ointment and the white columns show the placebo control group. The error bars stretch one standard error of the mean to each side. *** *p* < 0.001.

In the study group 93% felt the urge to defecate, most within 10 min after administration of suppositories. In the placebo group 37% felt the urge for defecation after administration of suppositories (*P* = 0.001) (Figure 1, Table 1). In the study group 90% had bowel movement, most within 10 min, after administration of suppositories as compared to 33% in the control group (*P* = 0.001) (Figure 1, Table 1). The smell was more troublesome in the study group (67%) as compared to the placebo group (7%) (*P* = 0.001).

## 5. Discussion

This is the first time fish oil and their free fatty acid extracts have been studied and reported in the form of suppositories and ointments, intended for anal use.

Hemorrhoids, anal fissure and pruritus ani are all common benign anal diseases that rely on corticosteroid-based medication for their treatment [22,23,24,25]. These medications have limited efficacy and other alternative therapies are needed. 

Human toxicology studies have not shown any toxic effects of fish oils and fatty acids, despite ingestion of up to 18 g/day of EPA in ethyl ester form [20].

No toxicological effects were observed in studies where animals were given up to 2000 mg/kg body weight/day of fish oils [26]. The U.S. Food and Drug Administration recommend that the daily intake of the polyunsaturated fatty acids EPA and DHA should not exceed 3 g. In comparison, assuming 100% absorption in the rectum, a person would have to use at least 12 of our lipid suppositories per day to reach that same amount. 

No toxic skin reactions were observed in this trial leading us to conclude that the 30% ointment is well tolerated in the studied dosage. There were only minor complaints of itching and pain in both groups with no statistically significant difference. There were four complaints about mucus secretion in the study group. All of these complaints were of minor concern to participants as the participants were not certain whether it was a melted suppository or real mucus fluids excretion. In all accounts the amount of fluid observed were small and only noticed soon after administration in conjunction with defecation. There were three complaints of seeing blood after defecation in the study group, although the participants were not certain whether this was actually blood or a mixture of feces and melted suppositories. In all instances this was a one time observation with only small traces, and there was no pain or other accompanying symptoms. The smell of the lipid suppositories was bothering to some of the subjects, but only during the time of administration of the medication and at defecation. One cannot rule out the possibility that this would have affected the objective parameters such as urge to defecate and bowel movements when noted during the time of administration of the suppositories.

The suppositories tested in this study clearly stimulated bowel movement causing defecation without causing diarrhea, mucus secretion or any prolonged effect after defecation. In the study group, most bowel movements occurred within 10 min after administration of the suppositories and only few occurred 30 min or longer after administration. The exact mechanism behind the bowel stimulation is not clear. Spiller *et al.* showed that long chain fatty acids (oleic acid) stimulated unusual motor patterns in the ascending colon and acted as laxative [27]. Furthermore, the use of plant oils as a laxative is well known [20,28]. It is conceivable that the effect of the lipid suppositories could be due to nonspecific stimulation of rectal mucosa nerve endings leading to a peristaltic reflex, causing the urge for defecation observed in the study group, after administration thus acting in a similar way as Bisacodyl [29]. The lipid suppositories did not seem to act as a bulking agent as there was no significant excess fluid accompanying the stools. As with other oils, the lubricant effect of the lipids could play a role. 

## 6. Conclusion

The lipid suppositories and ointment tested in this study seem to be safe and have a distinct effect on bowel movements in healthy subjects. Clinical trials in patients with constipation problems and preparation for endoscopy in the rectum and sigmoid are indicated.

## Declare

This study was done in accordance with the standards, and with the approval of the National Bioethics Committee, the Icelandic Medicines Agency and the Data Protection Authority.

EudraCT No.: 2007-004601-10.
